# Effect of Caging on *Cryptosporidium parvum* Proliferation in Mice

**DOI:** 10.3390/microorganisms10061242

**Published:** 2022-06-17

**Authors:** Hannah N. Creasey, Wen Zhang, Giovanni Widmer

**Affiliations:** 1 Cummings School of Veterinary Medicine, Tufts University, North Grafton, MA 01536, USA; hannah.creasey@tufts.edu (H.N.C.); wen.zhang@tufts.edu (W.Z.); 2 Gerald J. and Dorothy R. Friedman School of Nutrition, Tufts University, Boston, MA 02111, USA

**Keywords:** *Cryptosporidium*, cryptosporidiosis, microbiota, constrained ordination, dysbiosis

## Abstract

Cryptosporidiosis is an enteric infection caused by several protozoan species in the genus *Cryptosporidium* (phylum Apicomplexa). Immunosuppressed mice are commonly used to model this infection. Surprisingly, for a pathogen like *Cryptosporidium parvum*, which is readily transmitted fecal-orally, mice housed in the same cage can develop vastly different levels of infection, ranging from undetectable to lethal. The motivation for this study was to investigate this phenomenon and assess the association between the severity of cryptosporidiosis and the fecal microbiota. To this aim, the association between severity of cryptosporidiosis and caging (group caged vs. individually caged) and between the microbiota taxonomy and the course of the infection was examined. In contrast to mice caged in groups of four, a majority of mice caged individually did not excrete a detectable level of oocysts. Microbiota α diversity in samples collected between three days prior to infection and one day post-infection was negatively correlated with the severity of cryptosporidiosis, suggesting a causal negative relationship between microbiota diversity and susceptibility to *C. parvum*.

## 1. Introduction

Cryptosporidiosis continues to be a significant cause of morbidity in infants, particularly in Sub-Saharan Africa, Latin America and South-East Asia. In low-income countries, infection with *Cryptosporidium* parasites is a leading cause of debilitating infant diarrhea [1]. *Cryptosporidium parvum* and *C. hominis* infect the gastro-intestinal (GI) tract causing transient-to-persisting diarrhea which does not respond to antibiotics or antiprotozoal drugs [2,3,4].

Because *Cryptosporidium* parasites are difficult to maintain in culture [5,6], animal models, particularly mice, play an important role in this field of research. An enduring limitation of the mouse model is unexplained mouse-to-mouse variation in the severity of the infection, even among mice housed in the same cage. Since *C. parvum* is highly contagious and is easily transmitted, the observation that mice in a same cage develop infections of vastly different severity [7,8], from inapparent to lethal, is intriguing. In addition to practical reasons for investigating this phenomenon, uncovering factors affecting the severity of cryptosporidiosis will advance our understanding of parasite-host interaction.

Studies on various eukaryotic enteric pathogens have examined the interaction between the gut ecosystem and the pathogen [9,10,11,12,13]. With respect to cryptosporidiosis, the rationale that the intestinal microbiota could influence the severity of the infection rests on several premises, primarily the intracellular location in the intestinal epithelium of the parasite’s replicative stages, the parasite’s dependence on the import of host cell metabolites [14,15], and the enterocytes’ dependence on products of bacterial fermentative processes [16,17]. The hypothesis that the gut microbiota modulates parasite proliferation has begun to attract attention. Initially, Harp et al. showed that a normal intestinal microbiota delayed the onset of *C. parvum* oocyst excretion by several weeks [18]. These authors also found that the resistance of mice to C. parvum can be increased by transferring intestinal mucosa from resistant animals to susceptible infant mice [19]. A protective role of the gut microbiota against cryptosporidiosis was also observed in neonatal mice [20,21]. A study on the effect of inosine monophosphate dehydrogenase inhibitors on cryptosporidiosis in mice detected an increase in *C. parvum* virulence in response to the drug. This effect was attributed to an alteration of the intestinal microbiota and to reduced epithelial cell turnover [22]. The effect of probiotics on the course of cryptosporidiosis was also investigated [7,23,24,25,26,27]. Several of these studies found a beneficial effect of a probiotic *Lactobacillus* cocktail or of certain *Lactobacillus* species on the severity of cryptosporidiosis in mice. Del Cocco et al. reported a beneficial effect of *Enterococcus faecalis* in mice infected with *C. parvum* [28]. Experiments conducted in this laboratory found that mice ingesting a commercially available probiotic cocktail developed a more severe infection [7], whereas dietary fiber led to a milder infection compared to mice consuming a fiber-free diet [8]. Consistent with these findings, a recent study found that microbiota perturbation with cloxacillin, an antibiotic related to penicillin, led to more severe cryptosporidiosis in mice [29]. However, the absence of such an aggravating effect when mice were treated with a different antibiotic indicates that depletion or perturbation of the microbiota by itself does not necessarily change the course of the infection. Observation of the reverse effect, of cryptosporidiosis on the intestinal microbiota, has also been reported [18,26,30,31], whereas others have failed to detect taxonomic changes five and seven days after infection with *C. parvum* [29]. 

To our knowledge, the effect of mouse-to-mouse interaction on cryptosporidiosis has not been investigated. Social interaction among group-housed mice has been shown to induce chronic stress [32,33], leading us to hypothesize that the aggravating effect of co-housing on cryptosporidiosis we are reporting here may be related to stress or other physiological changes linked to dominant-submissive hierarchical interaction between cagemates. Stress has been shown to trigger the release of hormones, neurochemicals and neuropeptides, which can negatively affect immune function [34] and could thus affect resistance to infection.

Here we report a series of experiments with immunosuppressed adult mice infected with *C. parvum* aimed at documenting and quantifying the effect of caging on pathogen proliferation. 16S amplicon sequencing of the fecal microbiota was used to evaluate if cohousing affects the taxonomic profile of the microbiota, whether the severity of cryptosporidiosis can be predicted from the profile of the microbiota, and to describe the effect of cryptosporidiosis on the fecal microbiota.

## 2. Materials and Methods

### 2.1. Mouse Experiments

CD-1 mice were purchased from Charles River Laboratories (Wilmington, MA, USA). Mice were between four and six weeks of age at delivery. Animal experiments were approved by the Tufts University Institutional Care and Use Committee under protocol G2021-115. CD-1 mice were used in experiments 35, 37 and 38. The mice used for each experiment were ordered separately and came from a different breeding facility. Experiment 40 used inbred B6J.C3-Sst1 mice (Jackson Laboratories, Bar Harbor, ME, USA). In four distinct experiments, a total of 26 mice were maintained with one animal per cage for the duration of the experiment. The remaining mice were caged in groups of 4 (Table 1). All animals were housed in sterile shoe-box size filter-top cages 27 cm × 17 cm × 20 cm in size. The cages contained sterile corn chip bedding, ambient temperature was maintained at 20 °C, and a light-dark cycle of 12 h was applied. On three to four occasions in the course of each experiment, during the period of peak oocyst shedding, mice were housed overnight in filter-bottom cages to facilitate the collection of fecal material. The transfer between cages with bedding and with a wire bottom did not change the groupings. All mice, regardless of the intensity of the infection, were transferred to wire-bottom cages for the same duration. Sterile wire-bottom cages contained an approximately 6 cm^2^ acrylic platform on which the animals could huddle. Starting on the day of delivery, sterile drinking water supplemented with 16 mg/l dexamethasone 21-phosphate disodium (Thermo Fisher Scientific, Waltham, MA, USA) was provided ad libitum [35]. Mice were fed standard sterile rodent chow containing 18% protein (Teklad 2018, Envigo, Indianapolis, IN, USA). Except for caging, mice were subjected to the same treatment, given water supplemented with the same concentration of dexamethasone and ate the same chow. 

Oocysts were purified on a 10–25% (*w/v*) step gradient of Nycodenz (Alere Technologies, Oslo, Norway) [36]. Mice were infected per os with a dose of 1–2 × 10^4^ purified oocysts suspended in 20–30 μL water. Oocysts were 28, 70, 23 and 14 days old for experiments 35, 37, 38 and 40, respectively, when administered to mice. The day the mice were infected is defined as day 0 post-infection. Negative time points, like day three post-infection, indicate the day preceding the infection.

Fecal pellets were collected from individual mice directly from the animal or by transferring mice individually to a 1-L plastic beaker for about 10 min. Pellets were stored at −20 °C until they were processed for DNA extraction.

### 2.2. Quantification of Fecal Oocysts

Fecal oocyst output averaged over the number of samples was used as a measure of the severity of cryptosporidiosis. Feces collected overnight, or freshly collected fecal pellets, were manually homogenized, and thin smears were dried and heat-fixed on microscope slides. Slides were stained with TB Carbolfuchsin KF (BD, Franklin Lakes, NJ, USA, cat.# 212518) and counter-stained with TB Brilliant Green (BD, #212523) [37]. Oocyst counts in 10 randomly chosen 400 × microscope fields were counted. The selected smears were counted four times (40 fields total) to assess the level of variation. In addition to microscopic enumeration, quantitative PCR [5,38] and flow cytometry [7,8,39] were applied to measure fecal oocyst concentration in selected samples and to evaluate the accuracy of the microscopic enumeration. Briefly, for cytometry, samples were strained through a 100-μm mesh, stained with oocyst-specific monoclonal antibody 5F10 [7], and fluorescent events were quantified on a forward scatter (FSC) vs. green fluorescence (FL1) scatter plot acquired with an Accuri C6 flow cytometer (BD, Franklin Lakes, New Jersey, USA). A total of 12 samples from experiment 35 were analyzed using flow cytometry. For these 12 samples, two estimates of oocyst concentration were obtained, based on microscopic enumeration and on flow cytometry. The Pearson correlation coefficient r comparing the values obtained with the two methods was 0.987, (n = 12, *p* = 2.95 × 10^−9^). We also quantified *Cryptosporidium* DNA in fecal samples using a TaqMan assay targeting the heat-shock protein 70 (cgd3_3440) with forward primer tctgaaggaatgcgaacaact, reverse primer gggtttgtgattgcttgtcttt and probe 56-FAM/tgggcagag/ZEN/attggttggtgaagt/3IABkFQ. This analysis was applied to fecal DNA samples from the 14 mice in experiment 38. After controlling for DNA concentration, a Spearman Rank correlation analysis showed that mean oocyst counts and mean *C. parvum* DNA concentration were significantly correlated (rs = 0.88, n = 14, *p* = 3.5 × 10^−5^). 

### 2.3. 16S Amplicon Sequencing, Quality Control and Bioinformatics

The V1V2 region of the 16S ribosomal RNA genes was amplified with primers 27F and 338R. Individually barcoded amplicons were combined in approximately equal concentration and sequenced for 300 cycles in a MiSeq instrument (Illumina, San Diego, CA, USA) operated by Tufts Genomics core facility. Sequences were filtered for sequence noise using the programs screen.seqs and pre.cluster as implemented in *mothur* [40]. To estimate the magnitude of technical variation in the sequence data, two DNA extracts were prepared from each of four fecal samples. The duplicate samples were amplified and barcoded individually. The weighted UniFrac distance [41] between these duplicated amplicons was 0.039 (experiment 38) and 0.151, 0.156, 0.174 (experiment 35). As an additional quality control, we verified that clustering was not an artefact of samples from different experiments being sequenced in different MiSeq reactions. Five experiment 38 samples sequenced together with experiment 37 samples clustered with the remaining experiment 38 samples and not with samples sequenced in the same library. In addition, we also verified that Shannon diversity was uncorrelated to amplicon DNA concentration (experiment 37, n = 35, *p* = 0.40). Operational Taxonomic Units (OTUs) were formed using a 3% sequence similarity cut-off based on the OptiClust method [42]. OTUs with an average of fewer than 1 sequence per sample were removed. Clustering was tested using ANOSIM [43] as implemented in *mothur* [40]. Bonferroni correction was applied for multiple comparisons. To identify bacterial taxa which differed significantly between experimental groups, LDA [44] was performed using program LefSe [45] as implemented in *mothur*. Canonical correspondence analysis (CCA) and redundancy analysis (RDA) were used to assess the impact of one or multiple independent variables (predictors) on the microbiota. The choice of the constrained ordination method depended on whether the OTU abundance data were best modelled by a linear or a unimodal model [46]. In these analyses, the OTUs represented the dependent variables. The pseudo-F statistic [47] was calculated to test the null hypothesis of no association between dependent and independent variables. CCA and RDA were performed in CANOCO, release 5.15 [46]. Shannon diversity was calculated using program summary.single in *mothur*. The Phi Coefficient of Association [48] was calculated in CANOCO.

## 3. Results

### 3.1. Effect of Caging

#### 3.1.1. Effect of Caging on Cryptosporidiosis

**Figure 1:**
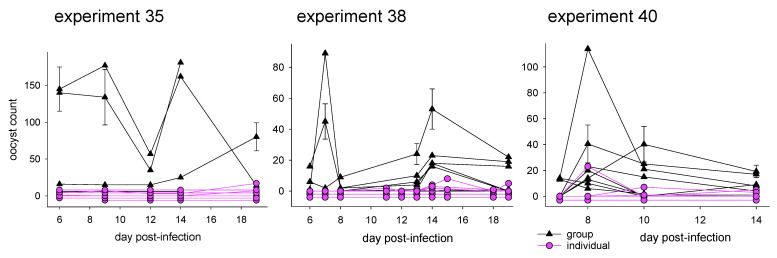
Oocyst shedding by mice housed in groups of four and in individually housed mice by experiment. Black lines indicate groups; purple lines indicate individual caging. Experiment 37 shedding curves are not shown because the grouped mice were not monitored individually. Selected error bars represent the standard deviation of four replicate counts. Counts of zero were artificially offset by two, three or four counts, depending on the *Y* axis scale, to reveal overlapping lines.

Mice housed individually were less likely to develop an infection (Table S1). Summed over the three experiments in which mice were monitored individually for oocyst excretion (Table 1), 95% (19/20) of mice caged in groups excreted oocysts. For the individually caged mice, the proportion of positives was only 58% (15/26). The association between caging and oocyst positivity was significant (Chi^2^ = 6.340, 1 d.f., *p* = 0.012). A similar effect of caging was observed when tallying individual observations, as opposed to individual mice. A total of 66% (68/103) of fecal samples from grouped mice were positive for oocysts. In contrast, for the individually caged animals oocysts were observed in only 30% (53/176) of samples. The association between caging and positive samples was also significant (Chi^2^ = 32.660, 1 d.f., *p* < 0.001). Looking at the oocyst counts, grouped mice excreted 56×, 18× and 6× more oocysts than individually caged mice in experiment 35, 38 and 40, respectively. The oocyst shedding curves for experiments 35, 38 and 40 (Figure 1) illustrate the magnitude and consistency of the caging effect.

#### 3.1.2. Effect of Caging on Fecal Microbiota

**Figure 2:**
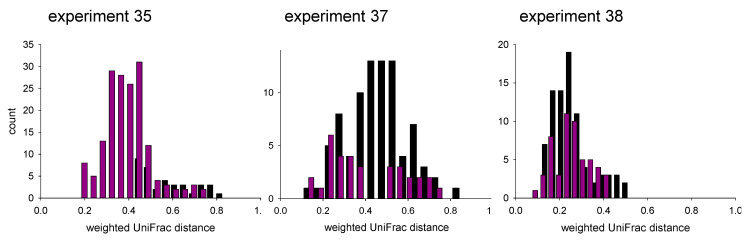
Effect of caging on microbiota β diversity between individual mice. Distribution of weighted UniFrac distances indicates that co-housing does not lead to microbiota convergence among mice. In experiment 35 the effect was the opposite, whereas in experiment 37 and 38, caging had no significant effect. Black bars indicate caged mice; purple bars indicate individually caged mice. *Y* axes show raw counts. *Y* axes are not drawn to scale.

Sporadic observations reviewed above indicate that *Cryptosporidium* proliferation may be affected by the intestinal microbiota and vice versa prompted us to investigate the effect of caging on the fecal microbiota, and the association between microbiota and the course of cryptosporidiosis. To assess the impact of caging, and specifically whether separate caging drove microbiota divergence, β diversity between pairs of samples collected from different mice was analyzed for experiments 35, 37 and 38 (Figure 2). The weighted UniFrac distance [41] was used as a measure of β diversity. To exclude the effect of temporal changes, only samples collected on the same day were compared. For experiment 35, which featured only one group of 4 jointly caged mice (Table 1), the number of pairwise β diversity values between mice caged jointly was 45. For individually caged mice, 168 diversity values were calculated for this experiment. For experiment 37, 85 distance values were calculated for grouped mice and 33 for individually housed mice. For experiment 38, 82 and 53, distance values were included for group-caged and individually caged mice, respectively. Caging only had a significant effect in experiment 35 (Mann-Whitney rank sum test; *p* < 0.001). In this experiment, contrary to expectations, co-housed mice harbored more divergent bacterial populations (mean UniFrac D = 0.55) as compared to individually caged mice (mean D = 0.39). For experiment 37, a mean weighted UniFrac distance of 0.45 (SD = 0.14, n = 85) and 0.40, (SD = 0.18, n = 33) was calculated for grouped and individually caged mice, respectively, which are statistically the same (*p* = 0.22). A similar conclusion was reached for experiment 38 as the mean weighted UniFrac distance was 0.25 for both treatment groups (group SDgroups = 0.09, n = 82; SDindividual = 0.08, n = 53). Together, these results show, somewhat surprisingly, that co-housing does not lead to microbiota convergence; to the contrary, in one instance co-housing appears to have driven microbiota divergence.

#### 3.1.3. Association of Cryptosporidiosis and Fecal Microbiota

The association between the severity of the infection with *C. parvum* and the fecal microbiota was examined for experiment 38. This experiment was selected because a complete 16S dataset and oocyst count series was available. Moreover, this experiment included two groups of jointly caged mice and no mice had to be euthanized before the end of the 19-day experiment (Table 1 and Table S1). RDA was used to test whether (a) mean oocyst count was a significant predictor of microbiota OTU profile, and (b) whether day post-infection was a significant predictor of OTU abundance. These analyses included 80 samples and 214 OTUs meeting the minimum abundance criterion described in Materials and Methods. For analysis (a), the effect of time (day post-infection) was removed by defining day post-infection as covariate, leaving oocyst count as the only predictor. RDA returned a highly significant association between oocyst output and OTU profile (pseudo-F = 3.7, *p* = 0.002). For analysis (b), where day post-infection was defined as predictor and oocyst output as covariate, the result was also significant (pseudo-F = 3.0, *p* = 0.004). We interpret the second result as evidence that the microbiota significantly changed over the course of the experiment. Oocyst output and day post-infection explained 4.6% and 3.8% of OTU variation in test (a) and (b), respectively. Thus, a large proportion of microbiota variation is associated with other, unknown variables. Indicating that in this experiment the association between oocyst output and microbiota was a consequence of the infection, rather than the microbiota impacting the course of the infection, RDA of the relative abundance of the 214 OTUs on day-3 and day 1 post-infection returned no significant association with oocyst output (pseudo-F = 1.0, *p* = 0.38, n = 27). Consistent with established observations [49,50], mouse weight gain was inversely correlated with mean oocyst output (Pearson r = −0.56, n = 14, *p* = 0.03). In light of these results showing that cryptosporidiosis impacts the intestinal microbiota, we further examined whether temporal changes in the microbiota profile were quantitatively related to the severity of the infection. In other words, we tested whether higher oocyst output was associated with a larger average β diversity experienced by each mouse’s microbiota over the course of the experiment. This analysis generated one datapoint for each of the 14 mice in experiment 38, where the distance value can be viewed as a measure of the distance in the multi-dimensional OTU space travelled by each mouse’s microbiota. A correlation analysis revealed that oocyst output and temporal change in the microbiota profile were not correlated, regardless of whether weighted UniFrac (Pearson r = 0.32, n = 14, *p* = 0.25) or unweighted UniFrac distance (r = 0.36, n = 14, *p* = 0.20) was used to quantify β diversity. Taken together, although the severity of cryptosporidiosis was found to be a significant predictor of the OTU profile, the magnitude of the temporal changes in microbiota taxonomy were not correlated to the severity of the infection.

### 3.2. Mice caged Individually

#### 3.2.1. Fecal Microbiota in Individually Caged Mice

**Figure 3:**
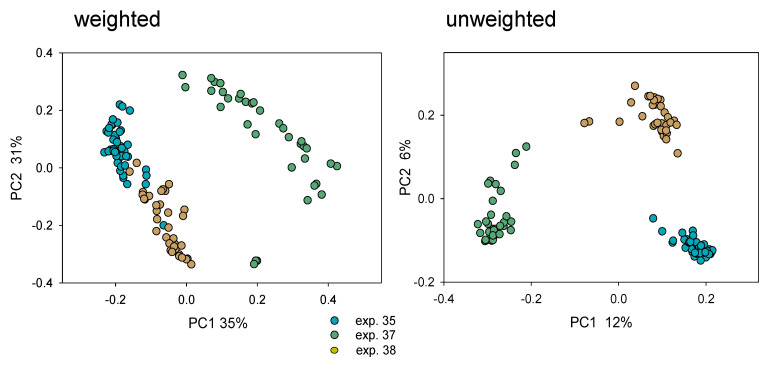
Mice in different experiments are populated with different intestinal microbiota. Mice for experiment 35 were purchased in July 2020, whereas experiments 37 and 38 were performed in February and March 2021. Each datapoint represents one fecal sample, regardless of the day of collection. Color indicates experiment; turquoise, experiment 35; olive, experiment 37; beige, experiment 38.

In the absence of mouse-to-mouse interaction, individually caged mice are an ideal model to measure the effect of specific experimental conditions on the course of cryptosporidiosis, on the intestinal microbiota, and to detect possible associations between infection and microbiota. The most striking observation related to the fecal microbiota obtained from 26 individually caged mice pertaining to three different experiments was the difference in microbiota composition between mice purchased at different times (Figure 3). This effect was apparent whether weighted or unweighted UniFrac was used to quantify β diversity. As expected from the PCoA plots, the effect of mouse lot on the entire set of 116 fecal samples collected at multiple pre- and post-infection timepoints from the 26 individually housed mice was highly significant based on both UniFrac metrics (ANOSIM, R = 0.75, *p* < 0.0001 and R = 0.85, *p* < 0001, weighted and unweighted, respectively). ANOSIM also revealed significant clustering for all three pairwise experiment comparisons. The R value for experiment 35 vs. 37 was 0.94, for experiment 37 vs. 38 0.97 and for experiment 35 vs. 38 0.74 (padjusted = 0.017). As apparent in Figure 3 and from these R values, β diversity between experiments was unrelated to the time elapsed between experiments. Experiments 37 and 38 were initiated within 37 days of each other, yet their microbiota were more divergent than between experiment 35 and 37 separated by 222 days. The observed segregation of the microbiota populating mice from different lots motivated us to investigate the possibility that clustering by experiment could be attributed to experimental conditions. Since different batches of oocysts were used in each experiment, bacteria contaminating the oocyst suspensions used to infect mice in each experiment could potentially affect the fecal microbiota. Arguing against this possibility, the clustering of samples collected between day −three and day one post-infection was also highly significant according to ANOSIM, including 39 samples collected during this early timeframe (R = 0.898, *p* < 0.0001). This outcome demonstrates that mice harbored very distinct microbiota from the onset of the experiment and that the segregation of microbiota by experiment is not a consequence of the oocyst inoculum.

To identify bacterial taxa which underlie the segregation of mouse microbiota by experiment, we calculated the phi coefficient of association [48] for each of the 375 OTUs using the experiment as classification (n = 116 samples). Eleven OTUs were highly diagnostic (Phi ≥ 0.9) for experiment 35 mice, 1 OTU was highly diagnostic for experiment 37 based on the same criterion, and 25 OTUs were diagnostic for experiment 38 (Table S2). This analysis showed the extent of taxonomic divergence underlying the β diversity shown in Figure 3.

#### 3.2.2. Association of Cryptosporidiosis and Fecal Microbiota in Individually Caged Mice

**Figure 4:**
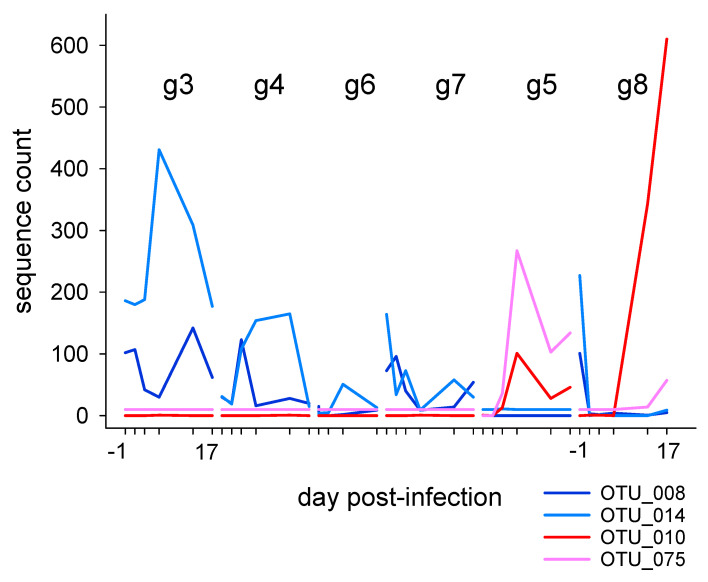
Temporal profile of relative abundance of OTUs significantly associated with low- and high-infection phenotypes in individually caged mice from experiment 37. Two OTUs shown in blue and light blue, respectively, are significantly more abundant in mice with low or no infection. Red and pink shows the relative abundance of sequences belonging to two OTUs significantly over-represented in heavily infected g5 and g8 mice. Tick marks on the *x* axis represent for each mouse day −one, one, three and 17 post-infection as indicated for mouse g3 and g8. The *y* axis scale represents the number of 16S sequences assigned to each OTU.

As described above in Section 3.1. and shown in Table S1, most mice caged individually excreted few or no detectable numbers of oocysts. The striking exception was the presence in experiment 37 of two out of six individually caged mice (g5 and g8) that were clearly infected and shed oocysts for 17 and 14 days, respectively. In an attempt to exploit this observation to identify factors explaining the different severity of cryptosporidiosis in this experiment, the fecal microbiota of the six individually housed experiment 37 mice was analyzed to address the following questions; (a) Can bacterial taxa predictive of the course of the infection with *C. parvum* be identified?; and (b) Did parasite multiplication in the GI tract affect the intestinal microbiota? The fact that these mice were housed individually removed a potentially confounding effect caused by the interaction between jointly caged mice. Because the 35 samples from the six experiment 37 mice could unambiguously be assigned to one of 2 groups, low and high infection, LDA was used. This analysis identified 73 OTUs out of 375, comprising a total of 72,767 16S sequences (48% of sequences in 375 OTUs) and two OTUs comprising 8424 sequences (6% of sequences) significantly associated with low and high infection, respectively. Figure 4 illustrates the temporal changes by mouse in the relative abundance of the two OTUs most positively and the 2 OTUs most negatively correlated with infection severity. The difference in relative abundance between OTU_010/OTU_075 (genus *Lactobacillus*) and OTU_008/OTU_075 (genus Lachnospiraceae_NK4A136_group; Table S3) in mice g5 and g8 vs. the four mice which excreted no oocysts is readily apparent. However, the abundance of these OTUs at the onset of the experiment is not associated with the outcome of the infection. For instance, in mouse g8, the abundance of OTU_008 and OTU_014 one day before infection (day −one) is high, which was not observed in mouse g5, the other mouse which also excreted oocysts. Consistent with this result, CCA focused on 19 samples collected before or within one day post-infection from individually caged mice from experiment 35, 37 and 38 did not reveal a significant association between the pre-infection microbiota and oocyst output (pseudo-F = 0.5, *p* = 0.95, n = 19, 375 OTUs). To account for the extensive microbiota β diversity between experiments (Figure 3), the effect of the experiment was removed from this analysis by defining this categorical variable as co-variate. To address question (b), namely whether parasite multiplication in the GI tract affected the intestinal microbiota, RDA was applied to the 35 samples from individually caged experiment 37 mice (all timepoints) using mean oocyst output as the independent variable. This analysis returned a highly significant association between oocyst output and OTU profile (pseudo-F = 14.8, *p* = 0.002). The analysis showed that a large fraction of microbiota variation (31%) is explained by oocyst output. RDA identified 15 OTUs out of 375 positively correlated with oocysts output, i.e., with a positive response variable score [51]. A clear difference was found between the class-level taxonomy of these 15 OTUs and the same number of OTUs most negatively associated with oocysts output, i.e., OTUs with the most negative response variable score (Chi^2^ = 22.9, 5 d.f., *p* < 0.001; Table S3). All OTUs in the latter category where classified in the class Clostridiales. Thirteen of these belong to various Lachnospiraceae genera. These OTUs can thus be viewed as indicators of undisturbed intestinal microbiota in experiment 37 mice. In contrast, OTUs at the positive end of the score range are taxonomically more diverse representing the phyla Actinobacteria, Bacteroidetes, Firmicutes and Proteobacteria. Taken together, whereas anaerobic species in the class Clostridia were inversely correlated with severity of cryptosporidiosis, a more diverse taxonomy including facultative aerobes was positively associated with the severity of the infection (Table S3). The presence of facultative aerobes is reminiscent of dysbiosis [16,52,53,54,55,56].

Similarly, as described above for experiment 38, the extent of temporal changes experienced by each mouse’s microbiota in response to the *C. parvum* proliferation was analyzed. To this aim, the UniFrac distance between samples collected from each mouse on subsequent timepoints was averaged over the duration of the experiment. As shown in Figure S1, the weighted UniFrac distance was uncorrelated with oocyst output (r = 0.156, n = 95, *p* = 0.13). However, unweighted UniFrac showed a modest, but significant, positive correlation (r = 0.26, n = 95, *p* = 0.01). Since unweighted UniFrac is sensitive to differences in the abundance of rare taxa, these results suggest that the infection impacts rare bacterial taxa.

**Figure 5:**
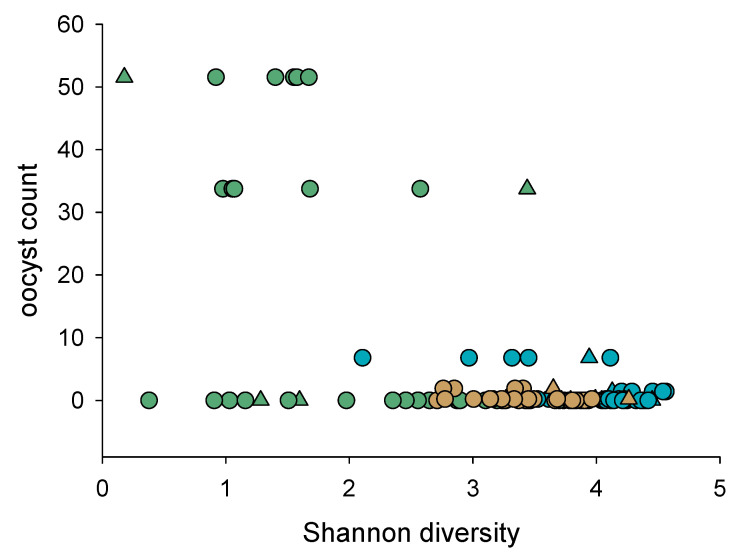
Severity of cryptosporidiosis is negatively correlated to α diversity. Oocysts were enumerated on acid-fast stained fecal smears as described in Materials and Methods and the intensity of the infection quantified by averaging oocysts counts over the number of observations. A total of 26 individually caged mice from experiment 35 (turquoise), experiment 37 (olive); and experiment 38 (beige) are included. Each datapoint represents one fecal DNA sample.

Consistent with *C. parvum* proliferation inducing dysbiosis, α diversity was negatively correlated with severity of infection (Figure 5). This correlation applies to all samples from experiments 35, 37 and 38 combined (r = −0.58, n = 112, *p* = 2.38 × 10^−11^). Significantly, α diversity of pre-infection samples was also negatively correlated with oocyst output (r = −0.54, n = 19, *p* = 0.02) (Figure S2). Although the correlation is low, the latter observation suggests that the native intestinal uninfected microbiota may affect the subsequent course of cryptosporidiosis.

## 4. Discussion

The observation that the native fecal microbiota populating mice ordered at different times from the same vendor can vary extensively is not new but deserves highlighting. Although not specifically discussed, similar variation can be inferred from a related study [30]. Microbiota segregation by breeding facility was also noticed by others as was the potential for this phenomenon to affect research reproducibility [57,58]. Unlike our observations, variation between microbiota from mice originating from different facilities assessed using a similar 16S protocol was caused by a relatively small number of OTUs unique to each breeding facility [58]. The cause of the extensive microbiota differences observed in our study is unknown, but is unlikely to be a consequence of post-delivery manipulations. Upon arrival, mice were transferred to sterile cages and given sterile food and water. Given the importance of the intestinal microbiota for a wide range of phenotypes [53,59,60,61,62], this observation supports the notion that the outcome of experiments with rodent models is affected by the source of the animals [63]. Populating germfree mice or pseudo-germfree mice [64] with a standardized microbial population may alleviate the problem and increase the experimental statistical power.

The research described here was designed to assess the effect of caging on *C. parvum* proliferation, on the microbiota, and to evaluate the interaction between the pathogen and the intestinal microbiota. The original motivation was extensive unexplained variation between jointly caged mice observed in a long series of experiments focused on understanding the impact of diet on the course of cryptosporidiosis [7,8]. Because of the specific focus of the research described here, all mice were infected with oocysts and no uninfected groups were included by design. This strategy precludes conventional comparisons between uninfected and infected animals. The resistance of most, but not all, individually caged mice to cryptosporidiosis enabled unplanned analyses to identify microbiota markers of susceptibility to *C. parvum*. The results are consistent with the view that there is no simple association between microbiota taxonomy prior to infection and susceptibility to cryptosporidiosis. How can this interpretation be reconciled with results described above showing that low microbiota α diversity tends to correlate with a more severe infection? The answer may be found in research showing that bacterial metabolites, rather than the abundance of bacterial taxa, impact enterocyte metabolism [65,66]. Because *Cryptosporidium* parasites multiply in intestinal epithelial cells, the taxonomy of the microbiota populating the intestinal lumen and the mucus layer may be less important than metabolites produced by intestinal microorganisms, such as short-chain fatty acids, which are known to affect enterocyte energy metabolism and immune functions [67,68,69]. Evidence consistent with this view has been reported in the context of research with *C. parvum* infected neonatal mice [70]. Further blurring the relationship between microbiota taxonomy and metabolic function is the fact there is no simple association between taxonomy and metabolism, as different bacterial taxa can have similar metabolism and vice versa [59]. A better understanding of the impact of the gut microbiome on parasite proliferation will likely require the translating of taxonomic information into function, either in silico [71] and/or using metagenomic and metabolomic analyses.

The unexplained mouse-to-mouse variation in the course of cryptosporidiosis in co-housed and individually housed mice led us to contemplate the possibility that the interaction between mice caged together could somehow affect the infection. Genetic heterogeneity among outbred CD-1 mice can be excluded as a cause of these observations since the same effect of caging was observed in experiment 40, which used inbred mice. As with other rodents, mice are considered social animals, and isolation is viewed as stressful [33]. The milder course of cryptosporidiosis we observed in isolated mice was therefore unexpected. Also unexpected was the variable outcome of the infection in mice housed in the same cage, particularly given that *C. parvum* is readily transmitted fecal-orally and the infectious dose in mice is low [72]. Together, these observations may reflect the effect of stress resulting from dominant/subordinate interaction among co-housed mice [33]. The same reasoning leads us to question whether isolation is actually stressful to mice. Stress-induced immunosuppression has been observed in mice infected with Herpes Simplex Virus [73]. Whether our observations can be explained by such immunological mechanisms is an open question, since mouse susceptibility to *C. parvum* requires immunosuppression [35,74,75]. Research with mice housed in enriched environments have investigated the effect of the social and physical environment on several disease phenotypes. Cao et al. [76] found evidence of cancer suppression in an enriched environment. The mechanisms mediating this effect were reported to involve the hypothalamus, the release of neurotrophic factors, and a resulting drop in serum leptin concentration. Coincidentally, leptin deficiency has been found to increase susceptibility to another enteric protozoan, Entamoeba histolytica [77]. Clearly, it is premature to speculate whether such mechanisms may be at play in *C. parvum* infected mice, but the fact that mice used in the experiments reported here were immunosuppressed argues against an immunological mechanism, instead pointing to pathways which are not affected by glucocorticoids. Other potential mechanisms linking caging conditions and susceptibility to cryptosporidiosis may relate to heat loss and hypothermia in individually caged animals. Ambient temperature below 30 °C has been reported to trigger huddling to reduce heat loss [78], a behavior precluded by individual housing. In our experiments, the effect of heat loss may have been accentuated by housing mice overnight in wire-bottom cages to enable the collection of feces. In the absence of body temperature measurements, it is not feasible to assess whether individually caged mice were hypothermic. Assuming individually caged animals were unable to maintain normal temperature, we are not aware of observations linking temperature stress to increased resistance to infection. To the contrary, hypothermia has been found to be predictive of mortality in mice infected with *Vibrio vulnificus* [79]. The effect of social stress on viral load was investigated in HIV infected macaques [80]. In this paper, they also analyzed the variability of multiple clinical variables. In contrast to the results reported here, Guerrero-Martin et al. found that among individually housed macaques, variability was higher. Chronic variable stress in rats was shown to affect the fecal microbiota and metabolome [81]. This observation raises the possibility that social deprivation caused by individual caging or chronic stress in cohoused mice affects the severity of cryptosporidiosis through the modification of the intestinal microbiota.

## 5. Conclusions

Replicate experiments with immunosuppressed outbred and inbred adult mice revealed a clear effect of caging on the course of cryptosporidiosis. The intestinal bacterial microbiota was found to have a modest, but significant, impact on the severity of the infection, while the perturbing effect of cryptosporidiosis on the microbiota was more pronounced. The research presented here is relevant to our goal of developing nutritional interventions to mitigate the debilitating effect of cryptosporidiosis in vulnerable infants [1,2,82] and immunodeficient individuals [83,84,85]. Such efforts are particularly relevant given the obstacles associated with developing and testing curative pharmaceuticals.

## Figures and Tables

**Table 1:** Summary of mouse experiments.

Exp.	Mouse Strain	Gender	Caging	Total Mice	*C. parvum* Isolate	Oocyst Counts	16S Sequence
35	CD-1	female	4/1/1/1/1/1/1/1/1	12	MD	yes	yes
37	CD-1	female	4/4/1/1/1/1/1/1	14	TU114	indiv. caged only	yes
38	CD-1	male	4/4/1/1/1/1/1/1	14	TU114	yes	yes
40	B6J.C3-Sst1	male	4/4/1/1/1/1/1/1	14	TU114	yes	no

Abbreviations: exp., experiment; indiv., individually.

## Data Availability

16S sequence data were deposited in FASTQ format in the Sequence Read Archive, National Library of Medicine, National Center for Biotechnology Information, under study accession numbers PRJNA841949 and PRJNA838969.

## Supplementary Materials

The following are available at: https://www.mdpi.com/article/10.3390/microorganisms10061242/s1. Table S1. Summary of oocyst enumeration in four experiments comprising mice caged in groups of four and individually caged mice. Within each experiment, shading is proportional to oocyst count. Fat horizonal line indicates that mouse was euthanized because moribund or because of other health issues. The ID of individually caged mice is colored as in Figure 2. Table S2. Indicator species in individually caged mice. Table S3. Taxonomic classification of 15 OTUs positively and negatively correlated with mean oocyst output in individually caged CD-1 mice from experiment 37. Red font indicates positive correlation, blue font, negative correlation. OTU, Operational Taxonomic Unit (97% sequence similarity); Resp.1, Response Variable Score 1 [51]. Figure S1. Severity of C. parvum infection impacts qualitative changes over time in microbiota composition. Figure S2. Severity of cryptosporidiosis is negatively correlated with microbiota α diversity prior to infection.
