# Supplementary figures and images for: Effect of Caging on Cryptosporidium parvum Proliferation in Mice
Source: Microorganisms. 2022 Jun 17;10(6):1242. doi: 10.3390/microorganisms10061242 (PMC9230662; doi:10.3390/microorganisms10061242)

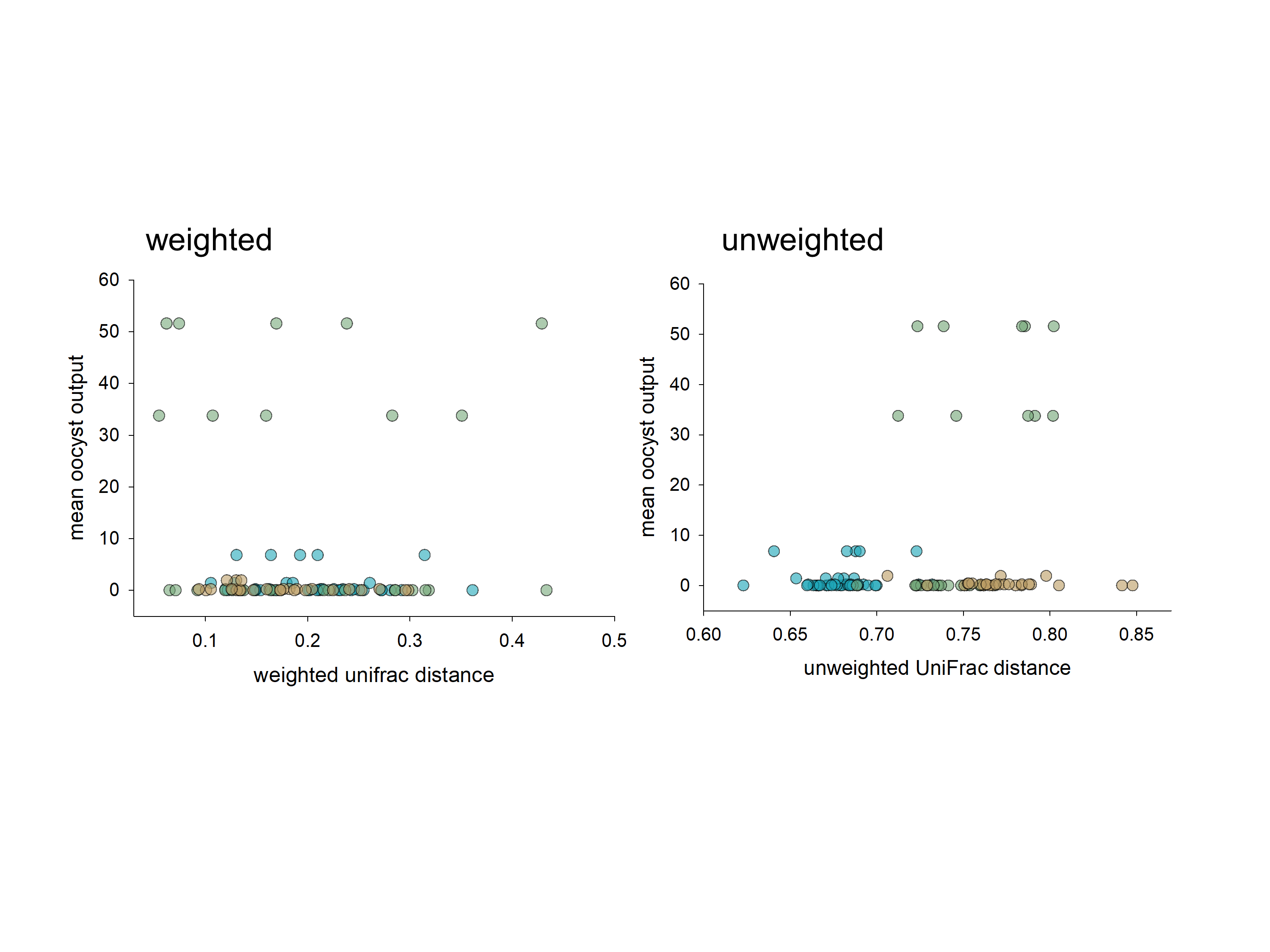

Supplement: Supplementary file 1 [file microorganisms-10-01242-s001.zip › Figure S1.tif]

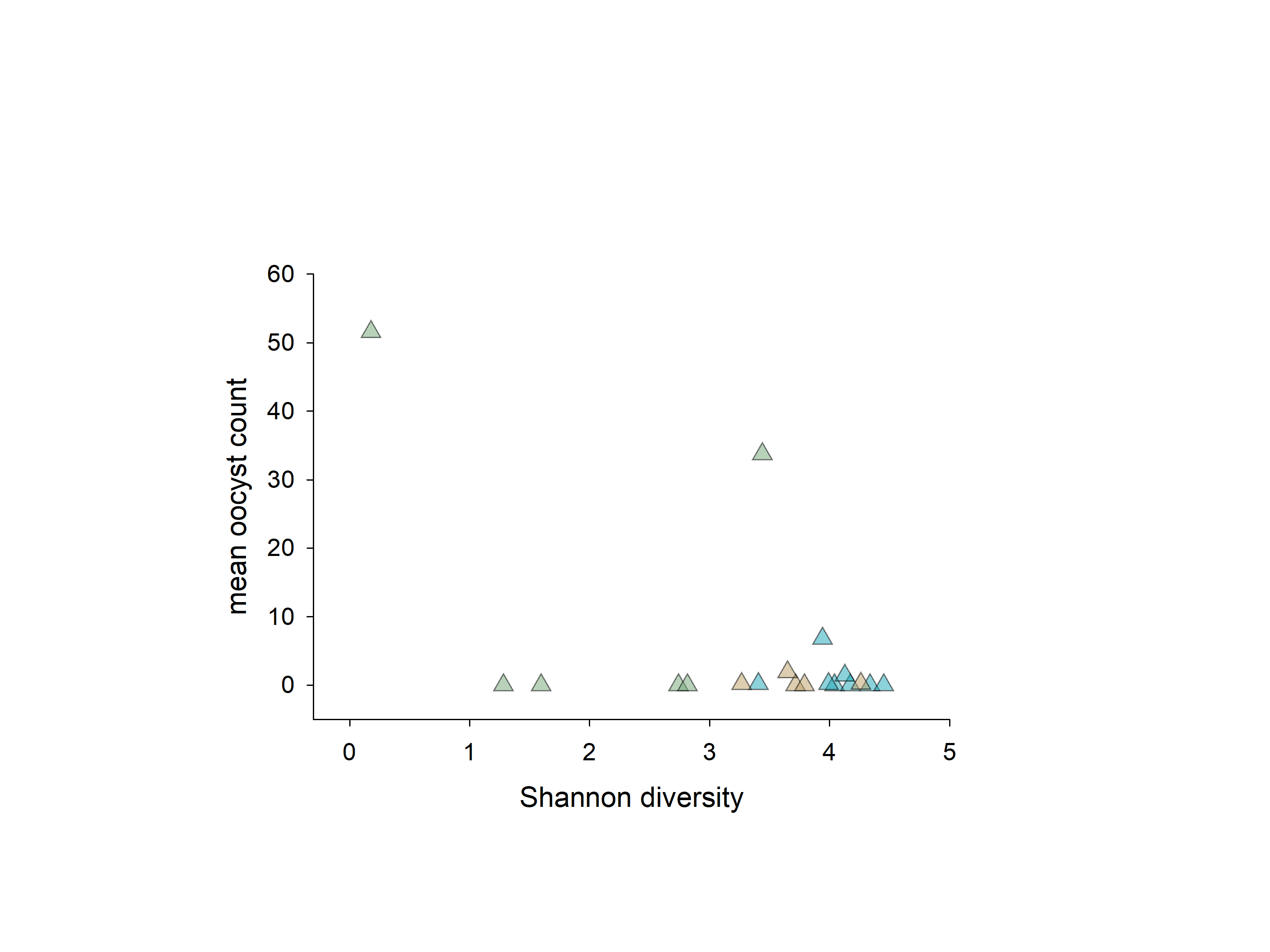

Supplement: Supplementary file 1 [file microorganisms-10-01242-s001.zip › Figure S2.tif]
